# Supplementary material for: Empty Sella Syndrome as a Window Into the Neuroprotective Effects of Prolactin
Source: Front Med (Lausanne). 2021 Jul 8;8:680602. doi: 10.3389/fmed.2021.680602 (PMC8295462; doi:10.3389/fmed.2021.680602)
Supplement: Supplementary file 1 [file Data_Sheet_1.pdf]

frontiers

SUPPLEMENTARY MATERIAL

S1. Additional clinical details for patient AJ and demographic information for all study participants.

Additional clinical details. Detailed review of the patient's clinical record is notable for the following narrative after initial diagnosis in 2009. Patient AJ was initially evaluated in the Multidisciplinary Pituitary Clinic at the University of Rochester for a large, 2.5 x 3.0 cm, prolactin-secreting pituitary tumor that demonstrated significant gadolinium contrast enhancement and expansion of the sella turcica - see Figure 1A. Her laboratory evaluation demonstrated an elevated prolactin level at 2523.91 ng/ml (normal reference range 4.8 – 23.3 ng/ml for non-pregnant females at our institution), confirming the diagnosis. No visual deficits were documented during this initial workup. Notable symptoms included daily bilateral frontal headaches, amenorrhea, mild cold intolerance, and a 20 lb. weight loss over the course of one year. In 2009 she was started on cabergoline 0.25 mg PO weekly, which was subsequently increased to twice weekly.

At her six-month follow up she reported a significant decrease in the frequency and severity of her headaches. At twelve months, MRI imaging demonstrated a dramatic response to treatment with very little evidence of adenomatous tissue within the sella and a mild ventral deviation of the optic chiasm. Her prolactin levels correspondingly decreased to within normal range (10.02 ng/mL) and she denied having any visual complaints. After two years, she concluded her initial round of treatment with stable imaging.

Seven months after discontinuing Cabergoline in 2010, the patient's headaches returned, and she began experiencing galactorrhea. While her laboratory workup was notable for a modest hyperprolactinemia (81.7 ng/ml), her imaging did not demonstrate any tumor progression. In fact, her MRI showed further descent of the optic chiasm and herniation of the third ventricle into the sella without any complaints of visual changes. She was restarted on Cabergoline at 0.25 mg twice weekly, and continued treatment for approximately seven years, with the exception of two brief periods off of medication. The first trial off of medication (2013) was for concern of medication side effects, which included postural instability, dizziness, anxiety, and panic attacks. The second trial followed a hysterectomy for endometriosis. Given that the primary indication for continued prolactinoma treatment in this patient was for galactorrhea, infertility, and amenorrhea, she no longer met criteria for treatment. Both times, she was unable to tolerate discontinuing the medication, with significant elevations in prolactin and the return of her

galactorrhea and headaches. Her headaches remained refractory to Topamax and Botox injections, and she was followed closely by our neurology headache colleagues. Consideration was given to decreasing the Prolactin dose to 0.25 mcg weekly, though the patient was reluctant to do so, given the closely perceived association between medication use and headache severity by the patient.

Her headaches were not well controlled, and 2 years later, her prolactin levels were again elevated to 164 ng/mL; thus she was restarted on cabergoline at 0.25mg once per week. She denied syncopal episodes, nausea, and galactorrhea. T2 weighted MRI with and without contrast did not show evidence of pituitary adenoma. Elevated prolactin levels were thought to be due to stalk effect.

Worsening daily headaches while on cabergoline prompted another MRI after 6 months, which showed no evidence of tumor recurrence; however, it was suggestive of optic chiasm herniation downward into the expanded sella, consistent with empty sella syndrome. The patient recalled visual changes dating back one year. She noted a decline in her peripheral vision but stated that her central vision had been stable. She did not report blurred vision, any decline in color vision, diplopia, or any transient complete loss of vision. She noted photosensitivity at night and bilateral ocular pain. Visual acuity was 20/20 OD and 20/20 OS. Ophthalmologic examination revealed heteronymous bilateral visual field defects. Prolactin levels were measured at 107.5 ng/ml. Normal OCT (optical coherence tomography) of the RNFL (retinal nerve fiber layer) indicated that the bitemporal hemianopsia is unlikely to be caused by residual chiasmal compression from the macroadenoma. She was instructed to reduce the cabergoline to 0.25 mg every other week. Her headaches remained well controlled while off of cabergoline during this period that overlapped with study observation.

Supplemental Table 1:

Basic Demographic information for study participants		
**Subject**	**Age**	**Sex**
AJ1	36	F
AJ2	36	F
AJ3	37	F
C1	57	M
C2	32	M
C3	30	F
C4	30	F
C5	30	F

Supplemental Table 1. Control participants were recruited from the greater Rochester, NY region with a mean age (35.8 yo) that approximates that of patient AJ (36 yo).

Supplemental Figure 1: Serum prolactin and Cabergoline Dose for patient AJ

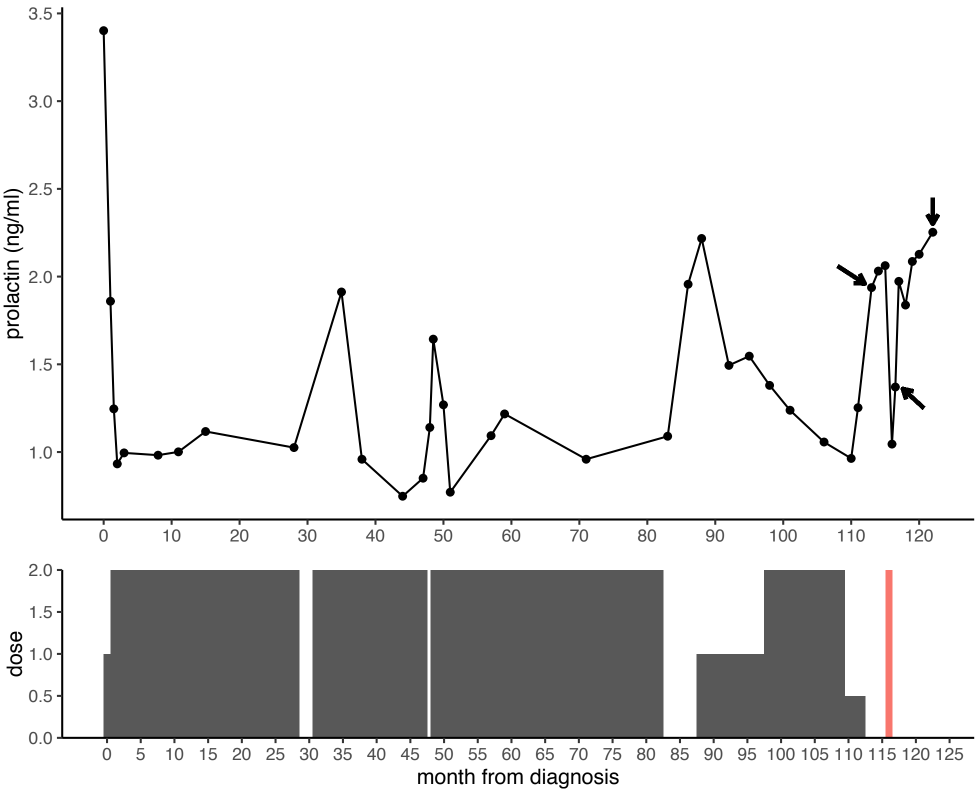

Supplemental Figure 1: Displayed are longitudinal measures of serum prolactin (top) as a function of Cabergoline dose (bottom) in patient AJ from the time of initial diagnoses in 2009 (month 0). Arrows represent prolactin levels obtained in conjunction with study visits (AJ1-3). The red line indicates cabergoline use against medical advice, overlapping with the second study visit (AJ2). Prolactin levels presented in base 10 log scale.

S2. Supplemental Diffusion MRI methods and analysis

Tractography was performed bilaterally, exclusively on the optic tracts, of all subjects. A single-voxel seed was deemed appropriate due to the small size of the optic tracts, consistent with methods previously published.[5] The seed was identified on the Eigenvector color map as the voxel with the highest fractional anisotropy value positioned 2-6 mm, or approximately 1-4 voxels, posterior to the optic chiasm (see Supplemental Figure 1). The optic chiasm was identified on the T1 image where the optic nerves meet, and seed identification began posterior the chiasm where the optic tracts start. Termination masks at the lateral geniculate nucleus, chiasm and cerebral peduncles functioned to regulate and direct fiber tracking. All seed and termination masks were checked for accuracy by three researchers (ES, AR, RH). 25,000 streamlines were sampled and the final probability distribution for each tract was thresholded at 2% of total streamlines. This means that voxels with less than 500 streamlines were excluded. This threshold was determined to be appropriate as it allowed for inclusion of a significant portion of the tract and also excluded extraneous voxels, using widely accepted white matter anatomy for comparison. Nevertheless, and because of the small size of the optic tracts, analyses were performed at several tractogram thresholds (0.5%, 1%, 2%, 5%, and 10%) to both confirm the appropriateness of previously published thresholds and ensure that effects are not related to over- or under-thresholding.[44] Radial (RD) and axial (AD) diffusivity were extracted for each tractogram and the resulting data imported into MATLAB for statistical analysis. These data were grouped by two variables - subject type (empty sella, control) and hemisphere (right, left); and plotted against visual measures organized by hemiretina as well as serum prolactin. See Supplemental Table 2 for data at each threshold.

Supplemental Figure 2: Approach to Tractography of the Optic Tracts.

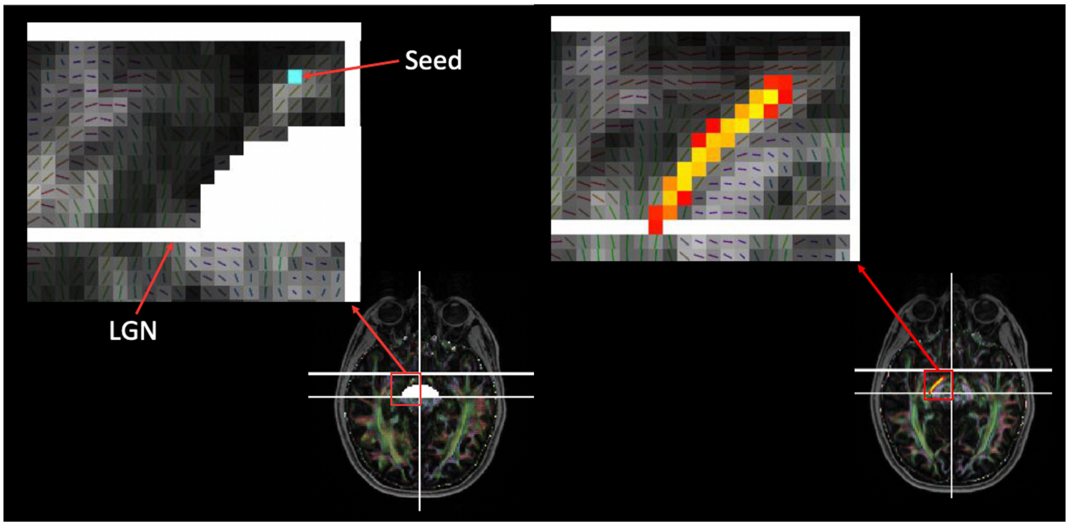

Supplemental Figure 2. (Left) Termination mask (white) and seed (cyan) input for probabilistic tractography. (Right) Thresholded probabilistic tractography result of the right optic tract in a healthy control (2% of streamlines, k = 500). Probability distribution is represented by red-yellow color gradient (red = 500 streamlines; yellow = 25,000 streamlines).

Supplemental Table 2:

Comparison of GCC thickness and optic tract diffusion metrics at different thresholds.			
**Diffusion Metric**	**% Threshold**	**R2**	***p* value**
FA	0.5	0.38	0.0327
	1	0.402	0.0268
	2	0.385	0.0313
	5	0.294	0.0686
	10	0.289	0.0717
AD	0.5	0.000561	0.942
	1	0.00314	0.863
	2	0.0256	0.62
	5	0.0414	0.526
	10	0.0645	0.426
RD	0.5	0.339	0.0469
	1	0.443	0.0181
	2	0.462	0.015
	5	0.381	0.0325
	10	0.304	0.0632
MD	0.5	0.238	0.107
	1	0.367	0.0369
	2	0.438	0.019
	5	0.389	0.0302
	10	0.285	0.0736

Supplemental Table 2: In order to verify the tractography method and choice of threshold, analyses were performed at several tractogram thresholds (0.5%, 1%, 2%, 5%, and 10%). Shown here are p values demonstrating the correlation between diffusion MRI indices of the optic tract and associated GCC hemifield, for each threshold.

Supplemental Table 3

Tract Averaged Diffusion Indices for all Study Participants								
**Subject**	**FA (right)**	**FA (left)**	**AD (right)**	**AD (left)**	**MD (right)**	**MD (left)**	**RD (right)**	**RD (left)**
AJ1	4.35E-01	4.16E-01	1.96E-03	1.80E-03	1.33E-03	1.23E-03	1.01E-03	9.47E-04
AJ2	2.71E-01	3.12E-01	1.87E-03	1.90E-03	1.46E-03	1.47E-03	1.26E-03	1.25E-03
AJ3	4.56E-01	4.07E-01	1.80E-03	1.97E-03	1.17E-03	1.37E-03	8.58E-04	1.06E-03
C1	4.69E-01	3.28E-01	1.75E-03	1.74E-03	1.13E-03	1.30E-03	8.28E-04	1.08E-03
C2	4.71E-01	3.96E-01	2.11E-03	1.83E-03	1.40E-03	1.27E-03	1.04E-03	9.87E-04
C3	4.88E-01	5.26E-01	1.98E-03	1.78E-03	1.25E-03	1.08E-03	8.84E-04	7.31E-04
C4	5.43E-01	4.45E-01	1.89E-03	1.77E-03	1.12E-03	1.14E-03	7.34E-04	8.33E-04
C5	2.61E-01	3.80E-01	1.89E-03	1.84E-03	1.48E-03	1.31E-03	1.28E-03	1.04E-03

Supplemental Table 3: Averaged diffusion values within each optic tract for each participant enrolled in the study.

Supplemental Figure 3:

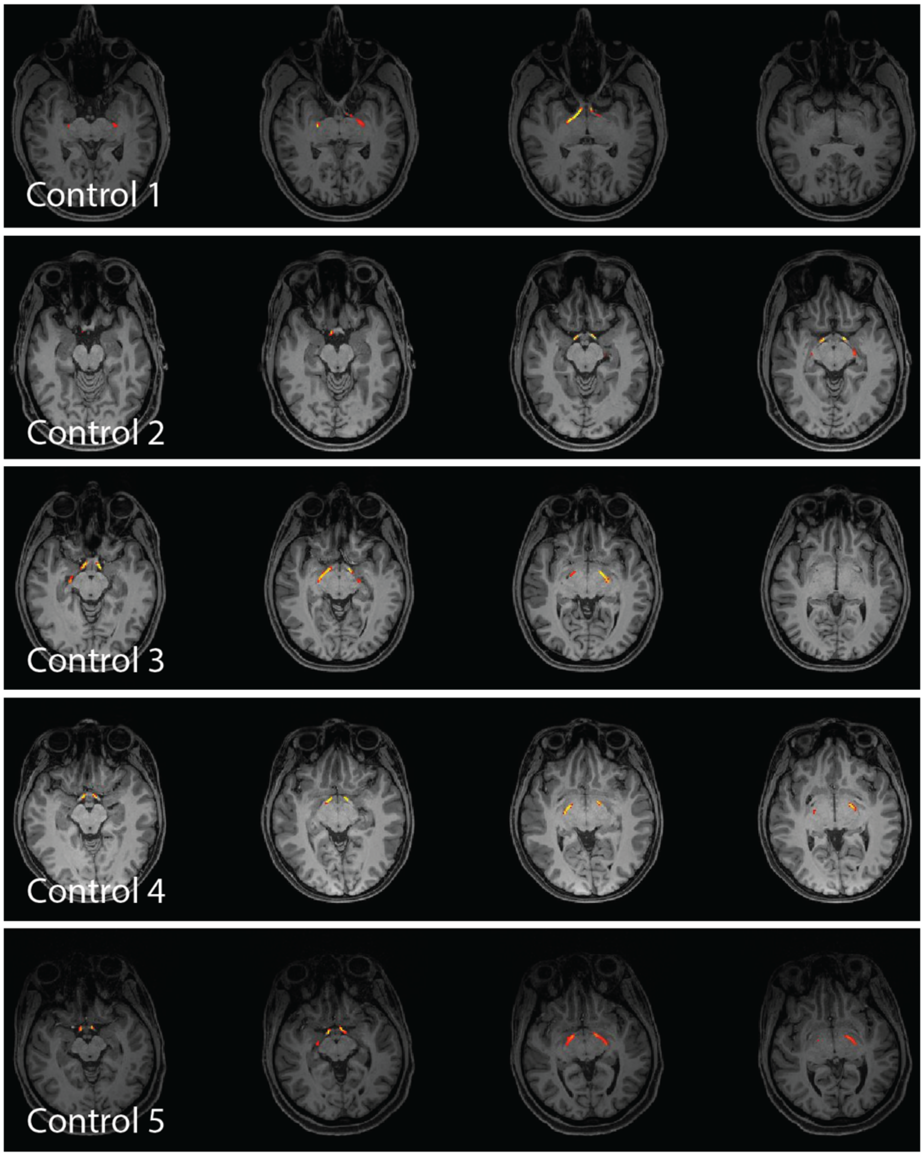

Supplemental Figure 3: Probabilistic tractography results of the optic tracts for each control participant.

Supplemental Figure 4:

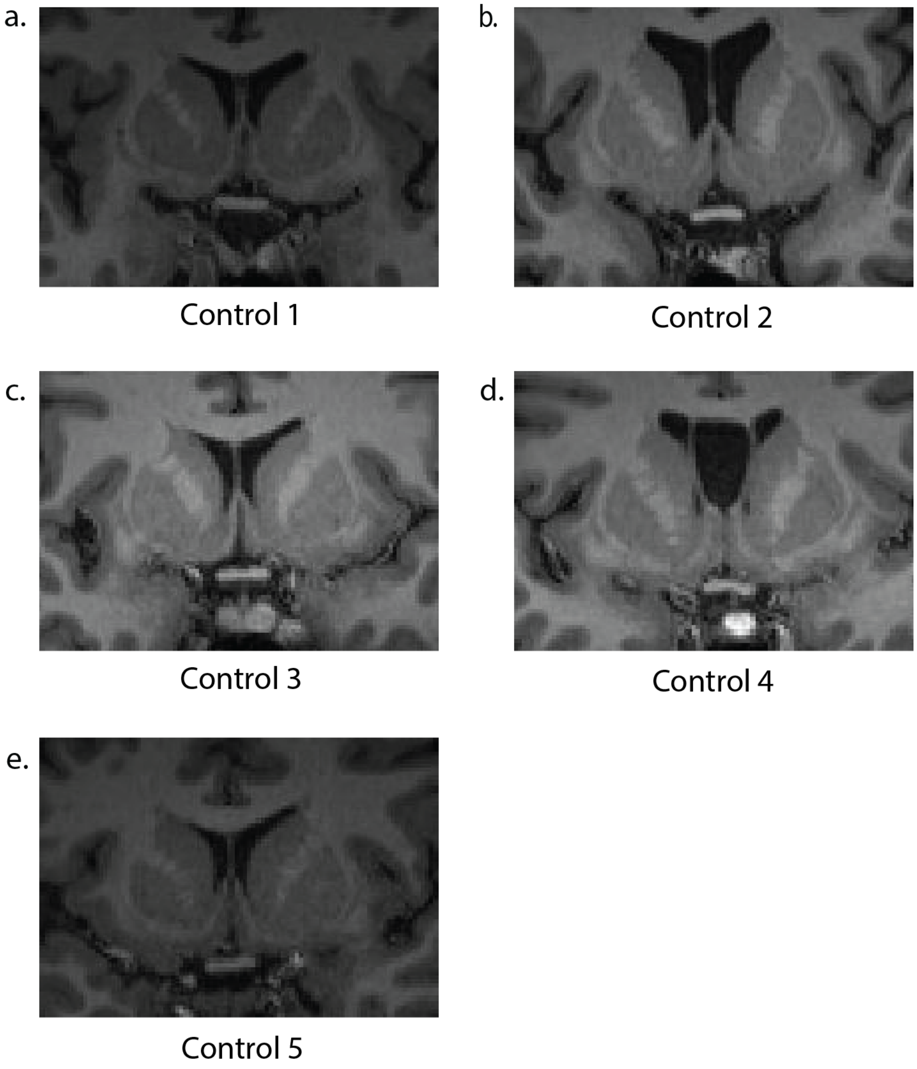

Supplemental Figure 4: T1-MPRAGE Coronal MRI images at the level of the chiasm for each control subject at the time of diffusion MRI scanning.

Supplemental Figure 5: Distribution of Streamlines for each Diffusion MRI Index.

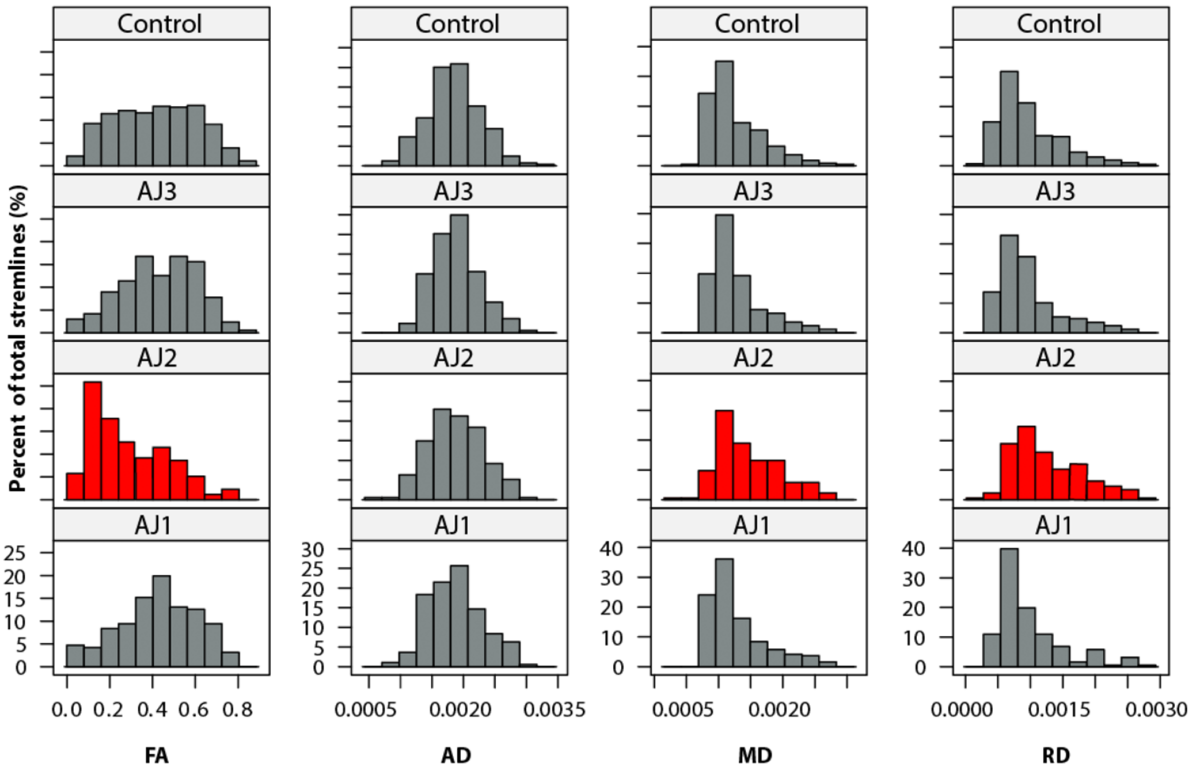

Supplemental Figure 5. Distributions of diffusion MRI metrics for the optic tracts in AJ at three time points (AJ1-3) and healthy control participants (n=10 total optic tracts). AJ scan 1 was completed after stopping cabergoline (high PRL); AJ scan 2 was completed after brief re-initiation of cabergoline (normal PRL); AJ scan 3 was completed 5 months after final termination of cabergoline treatment (high PRL). Diffusion metrics include axial diffusivity (AD), fractional anisotropy (FA), mean diffusivity (MD), and radial diffusivity (RD). Red denotes a statistically significant difference in streamline distribution compared with the control population (all $p < 0.05$). Notably, there was a significant increase in both radial and mean diffusivity, and a corresponding decrease in fractional anisotropy at AJ2.

S3: Additional Ophthalmologic Data

Supplemental Table 4

Individual ophthalmologic data for each study participant								
**Subject**	**OD Mean Deviation (dB)**	**OS Mean Deviation (dB)**	**OD M CST (µm)**	**OS M CST (µm)**	**OD RNFL (µm)**	**OS RNFL (µm)**	**OD GCC (µm)**	**OS GCC (µm)**
AJ1	-6.57	-6.15	231	240	75	88	69	74
AJ2	-9.51	-13.35	239	244	78	85	71	73
AJ3	-9.65	-6.05	236	246	78	88	71	75
Control 1	-1.33	-2.51	258	226	96	93	-	-
Control 2	-1.4	-0.49	316	317	91	97	73	78
Control 3	-1.81	-0.93	282	273	90	85	89	88
Control 4	-2.89	-3.09	277	275	104	103	82	80
Control 5	-2.54	-3.91	196	192	82	81	76	76

Supplemental Table 4: Retinal thickness measurements obtained from optical coherence tomography for healthy control participants (n = 10 total eyes) and patient AJ across all time points. AJ demonstrates a non-significant trend towards reduced thickness values for all retinal layers, consistent with values at the extreme bounds of normalcy, all $p > 0.05$ using Crawford and Howell's modified t-test. †Due to acquisition artifact, GCC thickness measurements were unable to be obtained for one of the 5 healthy controls; hence, control data are reported with n = 8 total eyes.

Supplemental Table 5

Mean Deviation reported by hemiretina for each study participant				
	Mean Deviation OD		Mean Deviation OS	
**Subject**	**Left hemiretina**	**Right hemiretina**	**Left hemiretina**	**Right hemiretina**
AJ1	-6.36	-8.63	-8.29	-5.93
AJ2	-10.39	-11.38	-18.17	-11.25
AJ3	-9.96	-13.71	-6.63	-7.29
Control 1	-1.68	-1.08	-2.13	-2.79
Control 2	-1.46	-1.38	-0.38	-0.82
Control 3	-2.11	-1.04	-0.67	-1.04
Control 4	-3.75	-2.33	-3.21	-3.11
Control 5	-3.46	-2.33	-4.29	-4.36

Supplemental Table 5: Hemiretina data derived from in-house MATLAB scripts, using raw Humphrey Visual Field data for all study participants.

Supplemental Table 6

Ganglion Cell Complex reported by hemiretina for each study participant				
	GCC OD		GCC OS	
**Subject**	**Right hemiretina**	**Left hemiretina**	**Right hemiretina**	**Left hemiretina**
AJ0	74.31	73.61	67.15	71.65
AJ1	75.24	74.02	67.90	73.46
AJ2	76.84	75.04	69.21	73.43
AJ3	-	-	-	-
Control 1	-	-	-	-
Control 2	77.04	79.48	79.59	66.45
Control 3	87.53	88.84	87.97	90.21
Control 4	79.20	80.56	81.53	81.59
Control 5	76.23	76.26	76.68	75.97

Supplemental Table 6: Hemiretina data for ganglion cell complex thickness derived using in-house MATLAB scripts.

Supplemental Table 7

Retinal Nerve Fiber Layer reported by hemiretina for each study participant				
	RNFL OD		RNFL OS	
**Subject**	**Right hemiretina**	**Left hemiretina**	**Right Hemiretina**	**Left hemiretina**
AJ0	74	59.2	78.2	83.6
AJ1	77.4	62.2	72.2	84
AJ2	80.8	61.2	73.6	93.4
AJ3	-	-	-	-
Control 1	89.6	88.6	90.2	81
Control 2	86	84.2	82	101
Control 3	103.6	70.4	60.8	104.2
Control 4	103.8	97.6	93.4	100
Control 5	76.2	76.8	67.4	80.2

Supplemental Table 7: Hemiretina data for retinal nerve fiber layer thickness derived using in-house MATLAB scripts.

Supplemental Table 8

Intraocular Pressure and Fundus Exam for all Study Participants						
	**Date**	**IOP Right Eye (mm Hg)**	**IOP Left Eye (mm Hg)**	**Cup to disc ratio Right Eye**	**Cup to disc ratio, Left Eye**	**Notes**
**AJ0**	10/08/2018	14	13	0.7	0.6	Optic disc cupping, no focal neuroretinal rim loss/ notch, no pallor both eyes, normal macula and periphery both eyes.
**AJ1**	03/08/2019	18	12	0.7	0.6	Optic disc cupping, no focal neuroretinal rim loss/ notch, no pallor both eyes, normal macula both eyes.
**AJ2**	07/10/2019	9	10	0.7	0.6	Optic disc cupping, no focal neuroretinal rim loss/ notch, no pallor both eyes, normal macula both eyes.
**AJ3**	11/13/2019	10	10	0.7	0.6	Optic disc cupping, no focal neuroretinal rim loss/ notch, no pallor both eyes, normal macula both eyes.
**Control 1**	02/25/2019	12	12	0.4	0.3	No disc pallor both eyes, normal macula and periphery both eyes.
**Control 2**	05/13/2019	17	16	0.2	0.1	No disc pallor both eyes, normal macula and periphery both eyes.
**Control 3**	06/03/2019	15	15	0.3	0.0	Myopic tilt, peripapillary atrophy both eyes, no disc pallor both eyes, normal macula and periphery both eyes.
**Control 4**	08/05/2019	14	13	0.6	0.6	Optic disc cupping with healthy neuroretinal rim both eyes, no optic disc pallor both eyes, normal macula and periphery both eyes.
**Control 5**	08/12/2019	20	15	0.3	0.3	No disc pallor both eyes, normal macula and periphery both eyes.
